# The Efficacy of Antioxidant Oral Supplements on the Progression of COVID-19 in Non-Critically Ill Patients: A Randomized Controlled Trial

**DOI:** 10.3390/antiox10050804

**Published:** 2021-05-19

**Authors:** Mahmoud M. A. Abulmeaty, Ghadeer S. Aljuraiban, Sumaya M. Shaikh, Naif E. ALEid, Lulwa R. Al Mazrou, Abdullah A. Turjoman, Mona S. Aldosari, Suhail Razak, Mervat M. El-Sayed, Tahani M. Areabi, Rokia M. Alsalafi, Yasser S. Al-Helio, Abdulrhman B. Almutairy, Haneen N. Molla

**Affiliations:** 1 Department of Community Health Sciences, College of Applied Medical Sciences, King Saud University, Riyadh 11362, Saudi Arabia; galjuraiban@ksu.edu.sa (G.S.A.); smarazi@ksu.edu.sa (S.R.); 2 Prince Mohamed Bin Abdulaziz Hospital, Riyadh 14214, Saudi Arabia; Shaikhs@pmah.med.sa (S.M.S.); aleidn@pmah.med.sa (N.E.A.); almazroul@pmah.med.sa (L.R.A.M.); turjomana@pmah.med.sa (A.A.T.); areabit@pmah.med.sa (T.M.A.); Alsalafir@pmah.med.sa (R.M.A.); alhelioy@pmah.med.sa (Y.S.A.-H.); Almutairya@pmah.med.sa (A.B.A.); 3 Clinical Nutrition Department, King Saud University Medical City, Riyadh 11472, Saudi Arabia; madosari@ksu.edu.sa (M.S.A.); hmolla@ksu.edu.sa (H.N.M.); 4 College of Food Sciences and Agriculture, King Saud University, Riyadh 11362, Saudi Arabia; melsayed@ksu.edu.sa

**Keywords:** COVID-19, antioxidants, cytokine storm, non-critically ill

## Abstract

Modulation of cytokine production using immunonutrition is a relatively novel concept to improve outcomes among patients with SARS-CoV-2 infection and is now hypothesized to help manage COVID-19, however, clinical evidence is lacking. This prospective, double-blinded, randomized parallel-controlled interventional clinical trial investigated the effect of antioxidant supplements on inflammatory cytokines and disease progression in non-critically ill patients. A total of 87 hospitalized COVID-19 patients were randomized using computer-generated-randomization into the supplement group (*n* = 18) and the placebo group (*n* = 16) for 10 days. Baseline and final nutritional screening via nutrition risk screening (NRS-2002) and subjective global assessment (SGA), as well as the recording of anthropometric, clinical, biochemical, and functional parameters, were done. Serum ferritin level, cytokine storm parameters such as interleukin-6 (IL-6), tumor necrosis factor-α (TNF-α), monocyte chemoattractant protein 1(MCP-1), C-reactive protein, total leukocyte count, lymphocytic count, and neutrophil-to-lymphocyte ratio were measured. Anthropometric and clinical parameters showed nonsignificant differences between groups. The hematology profile showed improvement in lymphocyte count in the supplement group. However, levels of alkaline phosphatase, IL-6, TNF-α, and MCP-1 were significantly lower in the supplement group. In conclusion, antioxidant oral supplementation significantly reduced the cytokine storm and led to partial improvements in clinical parameters among patients with non-critical COVID-19.

## 1. Introduction

The first case of coronavirus disease 2019 (COVID-19), the illness caused by the severe acute respiratory syndrome coronavirus 2 (SARS-CoV-2) virus, was reported in December of 2019 in Wuhan, China [1]. Just over a year later on April 12, 2021, 136 million people have contracted it and nearly 3 million have died from it, worldwide [2]. Several vaccines have recently been approved in many countries [3], however, less than 3% of the world’s population is fully vaccinated as of April 12, 2021, making treatment options a necessity.

COVID-19 can be asymptomatic, or it can demonstrate symptoms such as acute respiratory distress syndrome (ARDS). ARDS is a common symptom of COVID-19 and it is characterized by inflamed and stiff lungs, cough, shortness of breath, and breathing difficulties [4]. It is thought that much of the severity and death associated with COVID-19 ARDS is related to features of the cytokine storm and oxidative stress [5,6]. Oxidative stress is elevated during critical illness and may mediate the cytokine storm [7]. A cytokine storm is an excessive inflammation and disease affecting multiple organs caused by pro-inflammatory cytokine release from an unchecked immune response [5]. As such, there may be potential for treating symptoms of COVID-19 by targeting excessive inflammation. In addition to several pharmacological interventions that have been studied for treating cytokine storm syndrome in severe COVID-19 [5], there may be room for coadjutant therapies including dietary interventions [8].

Modulation of cytokine production using immunonutrition, or the modulation of the immune system by intervention with particular nutrients, is a relatively novel concept that has been applied to improve outcomes among the critically ill, surgical patients, and has now been theorized as a way to help tackle COVID-19 [9,10,11,12]. The use of antioxidants (e.g., vitamins C and E), which can counteract oxidative stress, has been well established as a therapy for ADRS, acute lung injury, and sepsis [13]. Under these circumstances, antioxidants have been associated with reduced time on ventilation, in ICU, and in hospital, as well as strengthened immune response, and reduced organ dysfunctions [13]. As a result, it is thought that antioxidants could also help patients with COVID-19 [13,14]. Although the role of antioxidants in COVID-19 treatment is not well established, some evidence of an association is available. In fact, several cross-sectional or prospective studies have shown that levels of various antioxidants and antioxidant trace elements (e.g., manganese, zinc, selenium, copper) were low among COVID-19 patients [15,16,17]. Notably, one study found that vitamin C levels among patients with SARS-CoV-2-associated ARDS were so low they were undetectable in 90% of patients examined [17]. One theory is that COVID-19 patients have depleted antioxidants due to their increased utilization to counteract the inflammatory response [15,17]. Thus, using antioxidants in high doses to account for their increased need may modulate the host immune response and ameliorate the cytokine storm associated with viral diseases such as COVID-19.

While some researchers have pointed to the potential for dietary supplements to prevent or treat COVID-19, they note that clinical research is needed to support such theories [8]. Very little clinical research has been conducted to look at the effects of antioxidants for treating COVID-19 and data from controlled trials are scarce due to the novelty of this disease. There have been a couple of case studies and an observational study looking at individual antioxidants (vitamin C) or antioxidant trace elements (zinc), demonstrating improved outcomes with administration, however, the small sample sizes introduce un-ignorable bias [18,19,20,21]. To our knowledge, no trials have been conducted looking at the effect of supplementation with a combination of antioxidants on COVID-19. Therefore, the current double-blinded randomized controlled trial (RCT) aimed to (1) identify the effect of anti-inflammatory-antioxidant oral dietary supplementation on the cytokine storm associated with SARS-CoV-2 infection; and (2) identify the effect of anti-inflammatory-antioxidant oral dietary supplementation on the clinical outcomes of patients with SARS-CoV-2 infection.

## 2. Materials and Methods

### 2.1. Subjects

**Figure 1:**
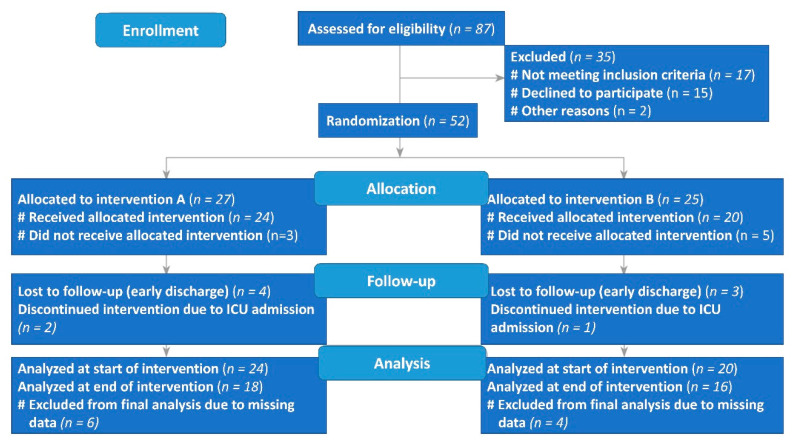
Flow diagram of the progress through the phases of the controlled randomized trial.

A prospective, double-blinded RCT was conducted between 30 December 2020 and 15 March 2021 in the Prince Mohamed Bin Abdulaziz Hospital, Riyadh, Saudi Arabia. Included were hospitalized adult patients (18–65 years) with SARS-CoV-2-positive PCR results with mild to moderate symptoms that mandate hospitalization (i.e., not requiring ICU admission). In addition, patients had to be on an oral diet, not pregnant or lactating women, not enrolled in another RCT, and receiving standard medical therapy of COVID-19 for inclusion in the study. Exclusion criteria included critically ill patients, COVID-19 patients on parenteral nutrition, or enrollment in another clinical trial. Of the 87 patients who underwent computer-generated randomization, a total of 35 were excluded (not meeting the inclusion criteria (*n* = 17), declined to participate (*n* = 15), other reasons (accepted then changed their mind; *n* = 2)). Therefore, 27 patients were assigned to the nutritional supplement (intervention A), and 25 patients were allocated to placebo (intervention B). Of the 27 patients assigned to receive intervention A, 18 received the nutritional supplement as assigned (Figure 1), while 3 did not receive the allocated intervention, 2 discontinued use due to intensive care unit (ICU) admission, and 4 were lost to follow-up (early discharge). In the placebo group, 16 patients received intervention B, while 5 did not receive the allocated intervention, 1 discontinued use due to intensive care unit (ICU) admission, and 3 were lost to follow-up (early discharge). Thus, a total of 34 adults completed the 10 days trial and were included in the final analysis. All participants signed a written consent after details of the study have been fully explained to them by a physician who belongs to the study team. The study protocol was approved by the central IRB committee of the Ministry of Health (MOH), Saudi Arabia, under reference number 20-#558E, dated: 27/12/2020 and registered in the clinical trial registry under reference NCT04323228 and the Saudi registry of a clinical trial under reference number SCTR No. 20092202.

### 2.2. Sample Size Calculation

According to a published study [22], the antioxidant/anti-inflammatory-oral nutrition supplement in sepsis patients produced a mean change in the IL-6, as a primary outcome, after 7 days compared to baseline of about 47.1 pg/mL in the intervention group vs. 3.3 pg/mL in the control group; α = 0.05; β = 0.20; δ = 43.8 pg/mL; s = 49 pg/mL. Therefore, 20 patients per group would be required using the following formula [23]:

(1) $$N=2\times {\left(\frac{z_{1^{-\frac{\alpha }{2}}}+z_{1-\beta }}{\delta }\right)}^{2}\times s^{2}$$

N = 2 × (1.96 + 0.845/43.8)^2^ × 492 = 19.69 ≈ 20 patients per group.

### 2.3. Study Protocol

All study participants were instructed to either ingest one capsule of a commercially available oral dietary supplement enriched with vitamins A, E, C, Zinc, and selenium (21st Century Antioxidant, Arizona, AZ, USA) or a cellulose-containing placebo capsule for 10 days. The supplements were served in labeled opaque red capsules of the same shape and color and were ingested in the morning after breakfast under the supervision of a nurse. The composition of the intervention supplement includes approximately: 1500 ug Vitamin A, 250 mg Vitamin C, 90 mg vitamin E, 15 ug Selenium, and 7.5 mg Zinc. The composition of the placebo was the same capsule volume containing 0.3 g dietary fiber cellulose powder (NutriCology Co, South Salt Lake, UT, USA). Baseline and final nutritional screening via nutrition risk screening (NRS-2002) and subjective global assessment (SGA), as well as the recording of anthropometric, clinical, biochemical, and functional parameters, were done.

### 2.4. Nutritional Screening and Assessment

Baseline and final screening for all participants were done using the (NRS-2002) [24], and SGA [25].

### 2.5. Anthropometric and Body-Composition Measures

Weight, height, mid-arm circumference (MAC), and triceps skinfold thickness (TST) were measured according to the protocol published by Abulmeaty et al., [26]. The body mass index (BMI) was calculated as weight in kilograms divided by the square of height in meters. The mid-arm muscle area (MAMA) and mid-arm muscle circumference (MAMC) were calculated according to Teo et al., [27] using the following equation:

{MAMA = (MAC − π × TST) 2/4π}, and MAMC = MAC − (π × TST)
(2)

Muscle function was also assessed by a handgrip dynamometer (JAMAR Hydraulic Hand Dynamometer, Performance Health, Warrenville, IL, USA). The average of two measures was used for the analysis [28].

### 2.6. Clinical Assessment

Daily records of peripheral O_2_ saturation (SPO_2_), body temperature, pulse, blood pressure, and respiratory rate were collected. Baseline and final readings were used for analysis. Participants requiring continuous admission in the ICU for ≥24 h were excluded from further investigation. For being more objective, a clinical severity scoring system was used to assess the severity of COVID-19 before and after intervention in both groups. Pandemic Respiratory Infection Emergency System Triage (PRIEST) was selected to assess the clinical severity of this study participants. The PRIEST-COVID-19 scoring system includes assessment of respiratory rate, SPO_2_, pulse, systolic blood pressure, temperature, alertness, need for oxygen therapy, gender, age, and performance status [29].

### 2.7. Biochemical Assessment

The biochemical parameters included complete blood count with differential counts of total leukocyte count, lymphocytic count, and neutrophil to lymphocyte ratio (Sysmex XP 300, Tokyo, Japan)). Liver function, renal functions, albumin level, creatinine level, alkaline phosphatase level, ferritin level, and C-reactive protein (Abbott ARCHITECT C4000 Automatic Biochemistry Analyzer). Cytokine storm parameters (interleukin-6, tumor necrosis factor-α, and monocyte chemoattractant protein 1) were analyzed by ELISA quantikine kits (R&D Systems, Abingdon, UK).

### 2.8. Statistical Analysis

The Statistical Package for the Social Sciences (SPSS) version 25 was used for the analysis. The descriptive statistics for continuous variables were presented as mean ± standard deviation, while other categorical variables were as percentages. The Shapiro–Wilk test was used to test the normality of study variables. The independent sample t-test was used for comparison between the supplement and placebo groups. For comparison of before and after repeated measures, a paired sample t-test was used. A chi-square test was used to compare the categorical variables between both groups. *p*-values < 0.05 were considered significant.

## 3. Results

### 3.1. Baseline Nutritional Characteristics of Study Groups

About 63.6% of the total patients were men. The mean age of patients was 45.08 ± 9.19 years in the supplement group and 52.80 ± 10.84 years in the placebo group. The baseline nutritional risk screening parameters and subjective global assessment parameters of study groups are shown in (Table 1). Scores of nutritional risk screening in 2002 were similar between groups, while scores of the subjective global assessment were significantly different between the supplement and placebo groups (*p* = 0.027); in the placebo group, there was more ‘very mild risk to well nourished’ patients compared to the supplement group, while more ‘mild to moderately nourished’ patients were in the supplement group compared to placebo at baseline (Table 1).

### 3.2. Anthropometric and Clinical Changes

**Figure 2:**
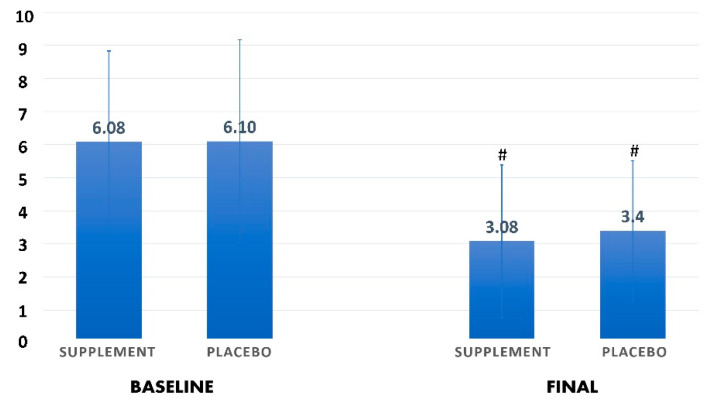
Changes in the clinical severity, based on PRIEST-COVID-19 clinical severity scoring system, before and after interventions in both groups. ^#^ indicates a significant difference compared to baseline scores in each group (*p* < 0.05).

The baseline anthropometric and clinical characteristics of patients who received the nutritional supplement and the placebo group are shown in Table 2. There were no important between-group differences in demographic characteristics, baseline anthropometric data, and other clinical features, except for pulse/minute reading, which was higher in the supplement group compared to the placebo group (*p* = 0.030). After 10 days, patients in the supplement group had a significantly lower body temperature (36.69 ± 0.41 °C) compared with baseline (37.28 ± 0.61 °C, *p* = 0.004), and respiratory rate (20.00 ± 1.26 b/min) compared with baseline (21.45 ± 1.97 b/min, *p* = 0.020). In the placebo group, pulse/minute reading was significantly higher (90.40 ± 15.92 compared with 77.80 ± 9.80 b/min) and RR was significantly lower (19.20 ± 1.32 compared with 21.00 ± 1.25 b/min) than baseline. No other significant differences were reported. The PRIEST-COVID-19 clinical severity scoring showed no significant difference between study groups at baseline and final assessment (Figure 2).

### 3.3. Hematological Changes

Baseline hematological characteristics of study groups are shown in Table 3. There were no important between-group differences in hematological characteristics, except for platelet reading, which was significantly higher in the placebo group compared to the supplement group (*p* = 0.034). After the intervention, there was a significant increase in WBC in the supplement group (8.06 ± 2.61 × 10^3^/uL) compared with baseline after 10 days (6.31 ± 2.10 × 10^3^/uL, *p* = 0.035). MCV, platelet count, and lymphocytes increased significantly in the nutritional group (88.64 ± 4.79 fL, 360.27 ± 156.99 × 10^3^/uL, 1.58 ± 0.86 × 10^3^/uL, respectively) after 10 days compared to baseline (90.08 ± 5.87 fL, 207.91 ± 68.26 × 10^3^/uL, 1.05 ± 0.55 × 10^3^/uL, respectively). Monocytes were significantly higher in the placebo group compared to the supplement group (*p* = 0.020) after treatment. No other significant differences were reported between the groups (Table 3). 

### 3.4. Biochemical and Inflammatory Changes

Table 4 shows the baseline biochemical and inflammatory parameters of the study groups. Albumin (g/L) level was significantly higher in the supplement group compared to the placebo group (*p* = 0.004). Potassium (mmol/L) level and MCP-1 (pg/mL) were higher in the placebo group compared to the supplement group (*p* = 0.040, 0.005, respectively). Other baseline biochemical data were similar between groups. After the intervention, AST significantly decreased in the supplement group (38.82 ± 23.66 U/L) compared to baseline (69.73 ± 39.73 U/L, *p* = 0.017) after 10 days of treatment. Additionally, C-reactive protein (CRP) significantly decreased in both groups compared to baseline, but there was no significant difference between groups after treatment. The supplement group had a significantly greater decrease in the monocyte chemoattractant protein-1 (MCP-1), Interleukin 6 (IL-6), tumor necrosis factor (TNF-α) (243.09 ± 37.80, 8.91 ± 4.23, 14.91 ± 5.32 pg/mL, respectively) than did the placebo group (375.33 ± 62.75, 11.89 ± 1.62, 21.11 ± 3.89 pg/mL, respectively) after 10 days of treatment. No other significant differences were reported between the groups.

### 3.5. Nutritional Status Changes

**Figure 3:**
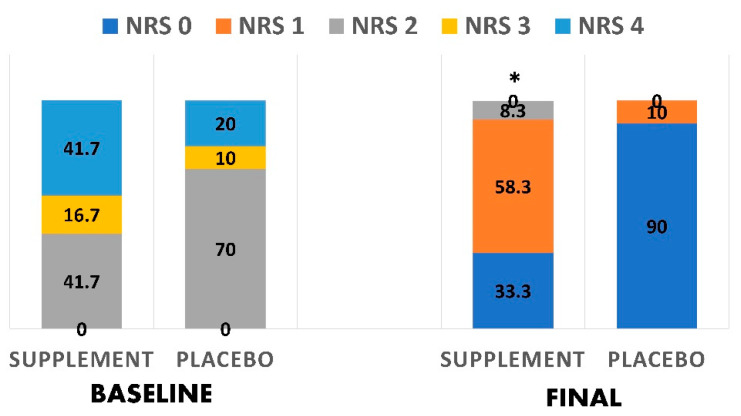
Changes in the nutritional status based on NRS2002 before and after interventions in both groups. * indicates a significant difference (*p* < 0.05).

**Figure 4:**
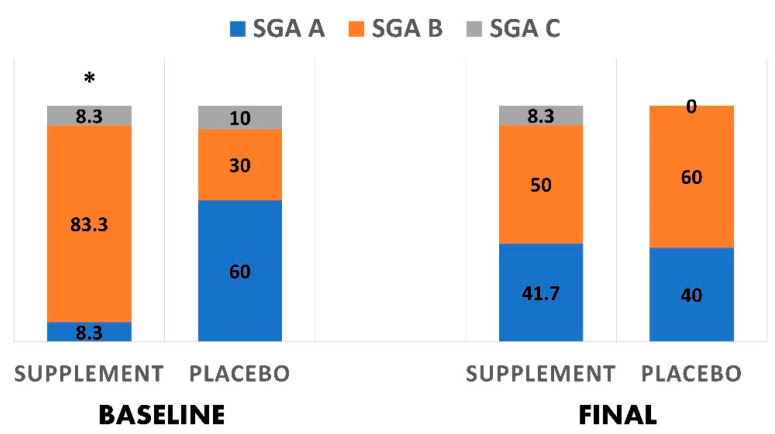
Changes in the nutritional status based on the subjective global assessment tool before and after interventions in both groups. * indicates a significant difference (*p* < 0.05).

Figure 3 shows the changes in the nutritional status across the two groups using the NRS2002. In the supplement group, there was a significantly greater decrease in those with NRS score 2 (41.7% vs. 8.3%), than in the baseline after 10 days of treatment, indicating an improved nutritional status. No other significant differences were reported between the groups. Furthermore, Figure 4 shows changes in the nutritional status using the subjective global assessment tool. In the supplement group, there was a significantly greater reduction in patients ‘mild to moderately malnourished’ after treatment (50.0% vs. 83.3%) than baseline. There was also a significantly greater increase in ‘very mild risk to well nourished’ patients (41.7% vs. 8.3%) when compared to baseline.

## 4. Discussion

The COVID-19 pandemic threatens patients, societies, and healthcare systems around the world and successful treatments are necessary for saving lives and conserving valuable hospital resources and ICU facilities. This research aimed to identify the effect of antioxidant oral dietary supplementation on the cytokine storm and clinical outcomes associated with infection with SARS-CoV-2. Our findings contribute to the growing evidence that the nutritional state of individuals with COVID-19 may affect the progression and outcome of the illness [9,10,11,12]. The results indicate that anti-inflammatory antioxidant oral dietary supplementation significantly dampens the cytokine storm and leads to partial improvements in some clinical parameters among patients with non-critical COVID-19. However, the improvement of clinical severity based on the PRIEST-COVID-19 score was not significantly different between the supplement and placebo groups.

Some of the most noteworthy findings of this study were the changes in the biochemical clinical parameters of COVID-19, including a significant decrease in aspartate aminotransferase in the supplement group (38.82 ± 23.66 U/L) compared to baseline (69.73 ± 39.73 U/L, *p* = 0.017) after 10 days of treatment. Additionally, the treatment group had a significantly greater decrease in several cytokines, including MCP-1, IL-6, and TNF-α (243.09 ± 37.80, 8.91 ± 4.23, 14.91 ± 5.32 pg/mL, respectively) than the placebo group did (375.33 ± 62.75, 11.89 ± 1.62, 21.11 ± 3.89 pg/mL, respectively). Aspartate aminotransferase and IL-6 are two of several biomarkers thought to be indicative of active cytokine storm syndrome with higher levels associated with severity in COVID-19 and/or with ARDS [5]. Therefore, the reductions seen in the present study are promising for treating COVID-19 symptoms and as a result, it may reduce the number of patients who require ICU admission and mechanical ventilation.

Despite double-blind randomization, there was a significant difference in SGA scores between the control and treatment groups at baseline, with the placebo group having more participants with better SGA scores than the treatment group. At the same time, there was no significant difference in the NRS2002 scores at baseline between the groups. The presence of differences at baseline with the SGA but not the NRS2002 concurs with research showing that although the two screening tools show good agreement with one another, there still tends to be significant differences in scoring [30]. Promisingly, there was a significant improvement in NRS2002 and SGA scores for the treatment group at the end of the study compared to baseline. This is especially noteworthy given that the nutrition status of patients based on the NRS2002 and SGA has been linked to clinical outcomes and mortality rates in hospitalized patients [31]. The improvements observed while patients in the present study were hospitalized are remarkable given the potential for nutritional status to wane during hospital stays [32]. Due to the acute nature of COVID-19, the nutritional status of the patients may not be affected right away but could deteriorate as the disease progresses due to impaired nutritional support and the development of the cytokine storm [15,17]. Thus, maintaining and supporting nutritional status during treatment is a critical aspect to consider in supporting recovery.

As the first trial to study the effects of a combination of antioxidants on COVID-19, comparing the findings to similar studies is challenging. Nevertheless, the results align with those from observational studies that found levels of individual antioxidants are low or lower in COVID-19 patients [15,16,17] and with small studies that suggest treatment with vitamin C or zinc was associated with improved COVID-19 outcomes [18,19,20,21]. Despite the limited research focused on COVID-19, the results from this study are in line with other research indicating that antioxidants can play a role in addressing ARDS and oxidative stress [13]. Recent reviews suggest reductions in the cytokine storm may be present with antioxidant supplementation, especially vitamin D [14]. Moreover, supplementation with zinc has been shown to have an inhibitory effect on the RNA-dependent RNA polymerase similar to that of SARS-CoV-2 [33]. Researchers have also stressed the importance of micronutrients in general for supporting the immune system for optimal resistance to infections [34]. Furthermore, the safety of antioxidant supplements for primary and secondary prevention as well as for therapeutic uses has been studied in meta-analyses [35]. Most studies examined as part of a meta-analysis found positive or null outcomes with the supplementation of various doses and combinations of antioxidants (e.g., beta-carotene, vitamins A, E), and only 3 of 66 RCTs had a negative (harmful) outcome in primary and secondary prevention studies [35]. Another meta-analysis also concluded there are potentially harmful effects (increased mortality) of antioxidant supplementation for primary and secondary prevention [36]. Therefore, the safety of using antioxidant supplementation is worth careful examination and consideration of the risks of not including it as a therapy option in future studies.

This study has some limitations and strengths worth noting. Firstly, although the participants were randomized into the two groups (treatment and control), there was an imbalance in the baseline measures for several variables that could have influenced the results. While randomization of patients to different treatments is left to chance, an imbalance between groups at baseline can lead to chance bias [37]. A larger sample size would presumably reduce the size of the imbalance at baseline, and future research with more participants can examine this theory [37]. On the other hand, randomization was one of the strengths of this study as was the double-blind nature of the research, which helps to eliminate differential treatment biases [38]. While this study fills an important research gap, it can also form the basis for several other studies on the subject that can further elucidate ideal combinations and dosages of antioxidants, treatment options for patients with more severe COVID-19 cases, cost-effectiveness, and the potential for antioxidant supplements to assist with not only treatment but also prevention.

## 5. Conclusions

This study fills an important gap in the literature. Given the novelty of the COVID-19 disease, few trials have been conducted looking at the effects of antioxidants as a coadjutant therapy option and none, to our knowledge, have looked at the administration of a combination of antioxidants and antioxidant trace elements. In light of the speed at which the SARS-CoV-2 infection has traveled the globe and the millions of deaths associated with it, treatments to help reduce severity are needed. The present study found that anti-inflammatory antioxidant oral dietary supplementation significantly dampened the cytokine storm and lead to partial improvements in clinical parameters among patients with non-critical COVID-19. Such promising findings can support updates to clinical protocols and inform future research.

## Figures and Tables

**Table 1:** Baseline nutritional risk screening parameters and subjective global assessment parameters of study groups.

Variables	Supplement (*n* = 24)	Placebo (*n* = 20)	
	% within Variable (% within the Group)	% within Variable (% within the Group)	*p*-Value
Gender			0.675
Female	62.5 (41.7)	37.5 (30.0)	
Male	50.0 (58.3)	50.0 (70.0)	
Scores of nutritional risk screening 2002 *			0.410
Score 2	41.7 (41.7)	58.3 (70.0)	
Score 3	66.7 (16.7)	33.3 (10.0)	
Score 4	71.4 (41.7)	28.6 (20.0)	
Scores of subjective global assessment **			**0.027**
A	14.3 (8.3)	85.7 (60.0)	
B	76.9 (83.3)	23.1 (30.0)	
C	50.0 (8.3)	50.0 (10.0)	

* NRS2002 of score ≥ 3 means malnourished. ** A: Very mild risk to well-nourished, B: Mild to moderately malnourished, and C: Severely malnourished. *p*-values < 0.05 were made bold.

**Table 2:** Anthropometric and clinical parameters before and after intervention in both study groups.

Variables	Supplement (*n* = 18)			Placebo (*n* = 16)			*p*-Value **	*p*-Value ***
	Baseline Mean ± SD	Final Mean ± SD	*p*-Value *	Baseline Mean ± SD	Final Mean ± SD	*p*-Value *		
Weight (kg)	**80.97 ± 20.16**	80.04 ± 17.34	0.775	85.60 ± 19.57	83.59 ± 19.01	0.172	0.577	0.644
BMI (kg/m^2^)	29.22 ± 4.99	28.85 ± 3.53	0.753	30.93 ± 7.64	30.21 ± 7.54	0.166	0.462	0.509
MAC (cm)	33.27 ± 3.28	33.09 ± 3.56	0.785	32.95 ± 5.60	31.65 ± 4.85	0.165	0.927	0.483
TST (mm)	27.27 ± 4.03	26.55 ± 6.33	0.650	24.40 ± 8.88	23.90 ± 11.86	0.858	0.372	0.541
MAMC (cm)	24.70 ± 2.76	24.75 ± 3.57	0.954	25.28 ± 3.79	24.14 ± 2.49	0.255	0.646	0.704
MAMA (cm^2^)	49.11 ± 10.68	49.66 ± 14.17	0.860	51.89 ± 16.50	46.82 ± 9.83	0.268	0.599	0.653
Temperature (°C)	37.28 ± 0.61	36.69 ± 0.41	**0.004**	36.88 ± 0.45	36.58 ± 0.23	0.165	0.156	0.473
Pulse (b/min)	87.91 ± 10.85	82.64 ± 7.89	0.147	77.80 ± 9.80	90.40 ± 15.92	**0.040**	**0.030**	0.148
SpO_2_ (%)	95.18 ± 1.99	95.18 ± 1.89	1.000	95.00 ± 2.00	95.20 ± 2.04	0.764	0.699	0.968
SBP (mmHg)	123.82 ± 18.05	121.18 ± 15.25	0.640	124.60 ± 8.49	125.40 ± 8.49	0.882	0.912	0.697
DBP (mmHg)	69.82 ± 8.24	73.73 ± 9.73	0.313	70.90 ± 8.05	79.90 ± 12.70	0.131	0.833	0.178
RR (b/min)	21.45 ± 1.97	20.00 ± 1.26	**0.020**	21.00 ± 1.25	19.20 ± 1.32	**0.027**	0.421	0.153
Hand grip (kg)	18.15 ± 7.66	20.50 ± 9.21	0.393	17.60 ± 7.92	22.55 ± 10.23	0.051	0.659	0.767

* Significance between variables before and after the intervention in each group; ** Significance between baseline variables in both groups. *** Significance between final assessment variables in both groups. *p*-values < 0.05 were made bold. MAC: Mid-arm circumference, TST: Triceps skin-fold thickness, MAMC: Mid-arm muscle circumference, MAMA: Mid-arm muscle area, SpO2: Saturation of peripheral O2, SBP: Systolic blood pressure, DBP: Diastolic blood pressure, RR: respiratory rate.

**Table 3:** Hematological parameters before and after intervention in both study groups.

Variables	Supplement (*n* = 18)			Placebo (*n* = 16)			*p*-Value **	*p*-Value ***
	Baseline Mean ± SD	Final Mean ± SD	*p*-Value *	Baseline Mean ± SD	Final Mean ± SD	*p*-Value *		
WBC (10^3^/uL)	**6.31 ± 2.10**	8.06 ± 2.61	**0.035**	7.98 ± 2.86	8.86 ± 2.18	0.433	0.119	0.422
RBC (10^3^/uL)	4.76 ± 0.47	4.70 ± 0.35	0.534	4.73 ± 0.62	4.77 ± 0.85	0.803	0.996	0.828
Hb (g/dL)	14.18 ± 1.37	14.06 ± 1.35	0.585	13.19 ± 1.67	13.55 ± 1.49	0.158	0.181	0.643
MCV (fL)	90.08 ± 5.87	88.64 ± 4.79	**0.041**	85.83 ± 8.36	85.89 ± 10.19	0.962	0.172	0.432
MCH (pg)	29.87 ± 2.02	29.72 ± 2.10	0.521	28.04 ± 2.94	28.95 ± 4.12	0.268	0.104	0.591
HCT (%)	42.81 ± 4.38	42.00 ± 3.44	0.251	40.42 ± 5.01	40.35 ± 5.13	0.942	0.288	0.580
Platelets (10^3^/uL)	207.91 ± 68.26	360.27 ± 156.99	**0.005**	318.40 ± 144.24	389.50 ± 120.23	0.147	**0.034**	0.868
Neutrophils (10^3^/uL)	4.85 ± 2.21	5.82 ± 2.44	0.167	6.07 ± 2.61	5.86± 1.71	0.822	0.227	0.973
Lymphocytes (10^3^/uL)	1.05 ± 0.55	1.58 ± 0.86	**0.008**	1.22 ± 0.38	2.09 ± 0.67	<**0.001**	0.498	0.135
NLR	6.29 ± 4.43	5.70 ± 5.46	0.664	5.59 ± 3.13	3.17 ± 1.81	**0.011**	0.796	0.190
Monocytes (10^3^/uL)	0.38 ± 0.17	0.47 ± 0.26	0.228	0.57 ± 0.31	0.79 ± 0.31	0.159	0.065	**0.020**
Basophils (10^3^/uL)	0.005 ± 0.005	0.006 ± 0.013	0.659	0.005 ± 0.007	0.002 ± 0.006	0.394	1.000	0.344
Eosinophils (10^3^/uL)	0.006 ± 0.015	0.026 ± 0.069	0.252	0.084 ± 0.131	0.055 ± 0.096	0.516	0.052	0.438

* Significance between variables before and after the intervention in each group; ** Significance between baseline variables in both groups. *** Significance between final assessment variables in both groups. *p*-values < 0.05 were made bold. WBC: White blood cells, RBC: Red blood corpuscles, Hb: Hemoglobin, MCV: Mean corpuscular volume, MCH: Mean corpuscular hemoglobin, HCT: Hematocrit value, NLR: Neutrophil lymphocytic ratio.

**Table 4:** Biochemical, inflammatory parameters, and cytokines levels before and after intervention in both study groups.

Variables	Supplement (*n* = 18)			Placebo (*n* = 16)			*p*-Value **	*p*-Value ***
	Baseline Mean ± SD	Final Mean ± SD	*p*-Value *	Baseline Mean ± SD	Final Mean ± SD	*p*-Value *		
ALT (U/L)	**63.36 ± 40.19**	65.00 ± 48.45	0.922	50.44 ± 20.21	58.44 ± 51.07	0.668	0.586	0.772
AST(U/L)	69.73 ± 39.73	38.82 ± 23.66	**0.017**	46.67 ± 17.03	30.44 ± 24.49	0.175	0.151	0.448
Albumin (g/L)	39.64 ± 4.86	41.64 ± 3.26	0.249	34.22 ± 2.49	40.31 ± 5.43	**0.007**	**0.004**	0.506
AlkP (U/L)	58.63 ± 15.45	56.25 ± 8.07	0.741	83.75 ± 15.20	110.50 ± 57.29	0.373	0.276	**0.020**
Total Bilirubin (umol/L)	7.59 ± 6.29	6.68 ± 2.26	0.713	5.35 ± 2.65	6.78 ± 2.19	0.377	0.834	0.943
Chloride (mmol/L)	103.00 ± 2.93	101.13 ± 2.53	0.294	103.00 ± 3.74	98.75 ± 4.11	0.178	0.373	0.238
Creatinine (umol/L)	72.55 ± 7.67	70.04 ± 11.69	0.299	78.66 ± 13.24	71.97 ± 17.98	0.222	0.201	0.896
Potassium (mmol/L)	4.19 ± 0.34	4.50 ± 0.56	0.138	4.98 ± 1.19	5.45 ± 2.02	0.650	**0.040**	0.225
Sodium (mmol/L)	138.88 ± 3.18	136.88 ± 3.36	0.159	134.75 ± 3.30	134.00 ± 2.94	0.718	0.271	0.178
Total Protein (g/L)	71.13 ± 6.62	70.50 ± 3.59	0.749	65.00 ± 5.94	68.75 ± 8.02	0.122	0.063	0.603
Urea (mmol/L)	4.95 ± 2.16	4.76 ± 1.56	0.819	7.08 ± 3.41	6.68 ± 2.32	0.789	0.108	0.118
CRP (mg/dL)	4.70 ± 3.85	1.28 ± 1.19	**0.049**	7.35 ± 6.47	1.44 ± 1.05	**0.033**	0.915	0.603
Ferritin (ng/mL)	619.2 ± 588.1	354.1 ± 318.9	0.160	386.2 ± 256.3	359.6 ± 233.4	0.190	0.513	0.979
MCP-1 (pg/mL)	565.73 ± 39.70	243.09 ± 37.80	<**0.001**	659.33 ± 64.29	375.33 ± 62.75	<**0.001**	**0.005**	<**0.001**
IL-6 (pg/mL)	22.09 ± 6.58	8.91 ± 4.23	<**0.001**	23.56 ± 7.06	11.89 ± 1.62	<**0.001**	0.692	**0.035**
TNF-α (pg/mL)	46.36 ± 3.20	14.91 ± 5.32	<**0.001**	47.11 ± 3.62	21.11 ± 3.89	<**0.001**	0.252	**0.003**

* Significance between variables before and after the intervention in each group; ** Significance between baseline variables in both groups. *** Significance between final assessment variables in both groups. *p*-values < 0.05 were made bold. ALT: Alanine aminotransferase, AST: Aspartate aminotransferase, AlkP: Alkaline phosphatase enzyme, CRP: C reactive protein, MCP-1: Macrophage chemotactic factor-1, IL-6: Interleukin 6, TNF-α: Tumor necrosis factor-alpha.

## Data Availability

Original data supporting these results are available on request from the corresponding author for reasonable purposes.
